# Risk factors and development of a prediction model for hematoma expansion in elderly patients with spontaneous intracerebral hemorrhage

**DOI:** 10.3389/fnagi.2026.1791268

**Published:** 2026-05-28

**Authors:** Xian-Xing Yu, Zhi-Yong Ye, Guang-Hui Zhu, Zhi-Hua Zhang

**Affiliations:** 1 Department of Emergency, Taizhou Hospital, Shanghai University of Traditional Chinese Medicine, Taizhou, Zhejiang Province, China; 2 Department of Neurosurgery, Taizhou Hospital, Shanghai University of Traditional Chinese Medicine, Taizhou, Zhejiang Province, China

**Keywords:** elderly, hematoma expansion, non-contrast computed tomography, prediction model, risk factors, spontaneous intracerebral hemorrhage

## Abstract

**Background:**

Hematoma expansion (HE) is a major determinant of early neurological deterioration after spontaneous intracerebral hemorrhage (sICH) in older adults. We aimed to identify independent risk factors for HE and to develop a predictive model for elderly patients with sICH.

**Methods:**

This retrospective observational study consecutively enrolled patients aged ≥60 years with sICH admitted between May 2023 and May 2024. Baseline non-contrast computed tomography (NCCT) and a follow-up NCCT within 48 h were required. HE was defined as an absolute increase in hematoma volume >6 mL or a relative increase ≥33%, with volumes quantified using the ABC/2 method. Candidate clinical, laboratory, and NCCT variables were screened with univariable logistic regression, then entered into multivariable logistic regression, and the variance inflation factor was assessed. Internal validation was performed using 1,000 bootstrap resamples.

**Results:**

Among 194 patients, 56 (28.9%) developed HE. Independent predictors of HE included age (adjusted odds ratio [aOR] 1.13 per year, 95% confidence interval [CI] 1.02–1.26; *p* = 0.025), National Institutes of Health Stroke Scale (NIHSS) score (aOR 1.21 per point, 95% CI 1.08–1.35; *p* < 0.001), neutrophil count (aOR 1.91 per 1 × 10^9^/L, 95% CI 1.34–2.73; *p* < 0.001), international normalized ratio (INR; aOR 1.70 per 0.1, 95% CI 1.08–2.69; *p* = 0.023), baseline hematoma volume (aOR 2.01 per 10 mL, 95% CI 1.34–3.02; *p* < 0.001), black hole sign (aOR 7.34, 95% CI 1.87–28.77; *p* = 0.004), and irregular shape (aOR 5.57, 95% CI 1.67–18.63; *p* = 0.005), whereas higher serum sodium was protective (aOR 0.76 per mmol/L, 95% CI 0.64–0.91; *p* = 0.003). The model showed an apparent area under the receiver operating characteristic (ROC) curve of 0.916 and an optimism-corrected AUC of 0.891.

**Conclusion:**

In elderly patients with sICH, HE might be associated with neurological severity, inflammatory burden, coagulation status, baseline hematoma volume, and adverse NCCT markers. The proposed model could provide strong discriminative performance and might support early risk stratification.

## 1 Introduction

Spontaneous intracerebral hemorrhage (sICH) remains a major cause of stroke-related mortality and long-term disability, particularly in older adults, who often have a greater comorbidity burden, more frequent pre-ICH antithrombotic exposure, and lower physiological reserve (Yan et al., 2023; Sadaf et al., 2021; Ueno et al., 2024). Despite advances in standardized acute ICH care, clinical outcomes remain highly variable, underscoring the need for practical tools for early bedside risk stratification (Parry et al., 2025; Hassan et al., 2024). Among early events after sICH, hematoma expansion (HE) is especially important because it is strongly associated with neurological deterioration and poor functional outcomes (Zhubi et al., 2025; Faghih-Jouybari et al., 2021). Because HE commonly occurs in the early hours after onset, timely identification of high-risk patients may help guide monitoring intensity, repeat neuroimaging, hemostatic treatment, and neurocritical care allocation (Yassi et al., 2024; Seiffge et al., 2025; Cindea et al., 2025b).

Prediction of HE requires integration of clinical, laboratory, and imaging data. Neurological severity scales, particularly the National Institutes of Health Stroke Scale, provide rapid assessment of early brain injury and may be useful for early prognostication in ICH (Kazaryan et al., 2024; Elkhatib et al., 2019; Sič et al., 2025). Laboratory abnormalities related to inflammation, coagulation, and electrolyte imbalance have also been implicated in HE. Furthermore, non-contrast computed tomography (NCCT), the first-line imaging modality in acute ICH, provides both hematoma volume assessment and morphological markers that may help identify unstable hematomas (Cindea et al., 2025a; Ji et al., 2025; Liu et al., 2025; Chen et al., 2025).

Although a growing number of studies have explored HE prediction after ICH, most have used mixed-age cohorts and increasingly rely on deep learning, radiomics, or other relatively complex multimodal approaches (Houssein et al., 2025; Zeng et al., 2026). Conversely, studies focused specifically on older adults have more often addressed overall prognosis rather than early HE (Hong et al., 2025). Since older patients have distinct comorbidity profiles, more frequent antithrombotic exposure, and reduced physiological reserve, it remains unclear whether a parsimonious model based solely on routinely available admission clinical, laboratory, and standard NCCT variables can provide practical early HE risk stratification for elderly patients with sICH (Stawarz et al., 2025). To address this gap, we aimed to identify independent risk factors for HE and develop a pragmatic prediction model tailored to this clinically vulnerable population.

## 2 Methods

### 2.1 Study design

This retrospective observational study consecutively enrolled elderly patients with sICH admitted to and treated at our institution from May 2023 to May 2024. Eligible patients were stratified into a HE group and a non-HE group based on the occurrence of HE on follow-up neuroimaging. The inclusion criteria were as follows: (1) age ≥60 years; (2) diagnosis of sICH confirmed by NCCT at presentation; (3) availability of a baseline NCCT obtained at admission and at least one follow-up NCCT within a prespecified time window sufficient to assess hematoma growth; and (4) complete clinical, laboratory, and imaging data required for risk factor analysis and model development. The exclusion criteria were: (1) secondary intracerebral hemorrhage due to trauma, ruptured aneurysm or arteriovenous malformation, intracranial neoplasm, hemorrhagic transformation of ischemic stroke, or other structural lesions; (2) primary intraventricular hemorrhage without parenchymal involvement; (3) prior neurosurgical intervention or early hematoma evacuation performed before follow-up imaging, precluding reliable assessment of HE; (4) absence of follow-up imaging or poor image quality preventing accurate hematoma quantification; and (5) incomplete key variables. Informed consent was obtained from all subjects and/or their legal guardian(s). The study was reviewed and approved by the hospital’s ethics committee and conducted in accordance with relevant guidelines and the Declaration of Helsinki. All data were anonymized before analysis to ensure participant confidentiality.

### 2.2 Hematoma expansion definition and imaging assessment

HE was defined as an increase in intraparenchymal hematoma volume on follow-up NCCT compared with baseline NCCT that met either of the following prespecified criteria: an absolute increase >6 mL or a relative increase ≥33%. Baseline NCCT was performed at admission, and follow-up NCCT for HE assessment was obtained within 48 h of the baseline scan (or earlier in the event of neurological deterioration) and before any surgical hematoma evacuation. Hematoma volume on baseline and follow-up scans was quantified using the standardized ABC/2 method. Briefly, A was the largest hematoma diameter (cm) on the axial slice with the maximal hematoma area; B was the largest diameter perpendicular to A on the same slice (cm); and C was calculated as the number of slices containing hematoma multiplied by slice thickness (cm), with partial-slice involvement weighted as 1 (>75% of maximal area), 0.5 (25–75%), or 0 (<25%). Hematoma volume was calculated as A × B × C / 2 and expressed in milliliters, assuming 1 cm^3^ approximately equals 1 mL.

### 2.3 Data collection

Demographic and clinical data were retrospectively extracted from the electronic medical record system for all eligible elderly patients admitted with sICH during the study period. Baseline variables included age, sex, body mass index, vascular risk factors, and medical history (hypertension, diabetes mellitus, atrial fibrillation, prior stroke), lifestyle factors (current smoking and alcohol use), and pre-ICH antithrombotic exposure (anticoagulant and antiplatelet therapy). Admission physiological parameters were obtained from the first documented vital signs in the emergency department or at hospital arrival, including systolic and diastolic blood pressure and heart rate. Neurological status at admission was assessed using the Glasgow Coma Scale (GCS) and the National Institutes of Health Stroke Scale, and the presence of consciousness disturbance was recorded based on the initial clinical examination.

Laboratory indices were obtained from the first blood sample collected within 24 h of admission, including complete blood count (white blood cell [WBC] count, neutrophil count, hemoglobin, platelet count), coagulation parameters (international normalized ratio [INR], prothrombin time [PT], activated partial thromboplastin time [APTT], fibrinogen), and biochemical tests (plasma glucose, serum creatinine, blood urea nitrogen [BUN], sodium, potassium). Inflammatory and fibrinolysis-related biomarkers (C-reactive protein [CRP] and D-dimer) were recorded when available within the same sampling window. For patients with multiple measurements within the predefined window, the earliest result was used in the analysis.

Imaging data were obtained from non-contrast head computed tomography performed at baseline and follow-up. Hematoma volume was quantified using the standardized ABC/2 method by two trained readers blinded to clinical outcomes, with discrepancies resolved by consensus. Hemorrhage location was categorized as lobar, deep, brainstem, or cerebellar. Predefined NCCT markers were assessed on baseline scans, including irregular shape, blurred margin, heterogeneous density, blend sign, and black hole sign, along with concomitant radiological findings (intraventricular hemorrhage, midline shift, and perihematomal edema). Inter-rater reliability for continuous hematoma volume measurements was assessed using the intraclass correlation coefficient (ICC), and agreement for categorical NCCT markers was evaluated using Cohen’s kappa coefficient. In cases of disagreement, the final imaging classification used for the primary analysis was determined by consensus. Follow-up computed tomography timing relative to the baseline scan and follow-up hematoma volume were recorded to determine HE status according to the prespecified volumetric criteria.

### 2.4 Statistical analysis

All statistical analyses were performed using IBM SPSS Statistics, version 26.0 (IBM Corp., Armonk, NY, United States). Continuous variables were assessed for normality using the Shapiro–Wilk test and visual inspection of the histogram. Normally distributed variables are presented as mean ± standard deviation and compared using Welch’s t test, whereas non-normally distributed variables are presented as median (interquartile range) and compared using the Mann–Whitney U test. Categorical variables are presented as numbers (percentages) and compared using the Pearson chi-square test or Fisher’s exact test, as appropriate. Inter-rater reliability for baseline and follow-up hematoma volume measurements was assessed using the ICC with 95% confidence intervals, and agreement for categorical NCCT markers was evaluated using Cohen’s kappa coefficient. Univariable logistic regression was used to screen factors associated with HE, and odds ratios (ORs) and 95% confidence intervals (CIs) were reported. Variables showing statistical evidence of association in univariable analyses and/or clinical plausibility were entered into a multivariable logistic regression model to identify independent predictors. Multicollinearity was assessed using variance inflation factors (VIFs), and backward stepwise selection was used to derive the final parsimonious model. Model discrimination was evaluated using the area under the receiver operating characteristic (ROC) curve, and the optimal cutoff probability was used to estimate sensitivity, specificity, positive predictive value, and negative predictive value. Subgroup analyses assessed discrimination across prespecified strata, including hemorrhage location, pre-ICH antithrombotic exposure, and follow-up computed tomography time window, with interaction terms used to assess effect modification. Sensitivity analyses examined robustness to alternative HE definitions and to the exclusion of extreme values. The events-per-variable (EPV) ratio was calculated as the number of HE events divided by the number of predictor parameters retained in the final multivariable model, excluding the intercept. Internal validation was performed using 1,000 bootstrap resamples, repeating the entire model-development process in each resample. Optimism-corrected AUC was calculated by subtracting mean optimism from the apparent AUC. Calibration was assessed using the calibration intercept, calibration slope, and Brier score. Clinical utility was evaluated using decision curve analysis (DCA) by comparing the net benefit of the model with the default treat-all and treat-none strategies at selected threshold probabilities. All statistical tests were two-sided, and *p* < 0.05 was considered statistically significant.

## 3 Results

### 3.1 Patient enrollment and study cohort

During the study period from May 2023 to May 2024, 219 consecutive inpatients presenting with sICH were screened for eligibility. After applying the prespecified inclusion and exclusion criteria, 25 patients were excluded for the following reasons: absence of follow-up NCCT within the 48-h assessment window after the baseline scan or inadequate image quality that precluded reliable hematoma volume assessment (*n* = 11); secondary intracerebral hemorrhage attributable to trauma, vascular malformation or aneurysmal rupture, intracranial neoplasm, or hemorrhagic transformation of ischemic stroke (*n* = 6); surgical hematoma evacuation or decompressive procedures performed before follow-up imaging, preventing standardized assessment of HE (*n* = 5); and incomplete key clinical or laboratory variables required for risk factor analysis and model development (*n* = 3). Accordingly, 194 patients comprised the final analytic cohort. Based on follow-up imaging, 56 patients were classified into the HE group and 138 into the non-HE group, yielding an overall HE incidence of 28.9% (56 of 194).

### 3.2 Baseline clinical characteristics

The HE group was older than the non-HE group (73.90 ± 5.37 vs. 70.83 ± 4.90 years; t = 3.71, *p* < 0.001) and had a slightly higher body mass index (25.18 ± 2.80 vs. 24.30 ± 2.81 kg/m^2^; t = 1.99, *p* = 0.049). Sex distribution showed a non-significant trend toward a lower proportion of men in the HE group (53.6% vs. 67.4%; χ^2^ = 3.28, *p* = 0.070). Among vascular risk factors, atrial fibrillation was more prevalent in the HE group (28.6% vs. 14.5%; χ^2^ = 5.22, *p* = 0.022), whereas the prevalence of hypertension, diabetes mellitus, and prior stroke did not differ significantly between groups (all *p* > 0.05). At admission, the HE group had higher systolic blood pressure (164.98 ± 21.08 vs. 155.88 ± 21.33 mmHg; t = 2.72, *p* = 0.008), diastolic blood pressure (97.23 ± 15.36 vs. 89.52 ± 12.55 mmHg; t = 3.33, *p* = 0.001), and heart rate (83.22 ± 11.99 vs. 78.32 ± 13.20 bpm; t = 2.50, *p* = 0.014). Neurological severity on presentation was greater in the HE group, with lower GCS scores (11 [10–13] vs. 13 [12–14]; Z = −5.34, *p* < 0.001), higher National Institutes of Health Stroke Scale scores (15 [8–17] vs. 8 [4–13]; Z = 5.16, *p* < 0.001), and a higher proportion of patients presenting with consciousness disturbance (51.8% vs. 31.2%; χ^2^ = 7.26, *p* = 0.007). Regarding pre-ICH antithrombotic exposure, prior anticoagulant therapy was more frequent in the HE group (23.2% vs. 9.4%; χ^2^ = 6.53, *p* = 0.011), whereas prior antiplatelet therapy did not differ between groups (*p* = 0.919; Table 1).

**Table 1:** Baseline clinical characteristics between groups.

Variable	HE (*n* = 56)	Non-HE (*n* = 138)	Test statistic	*p* value
Age, years	73.90 ± 5.37	70.83 ± 4.90	t = 3.71	<0.001
Men sex, n (%)	30 (53.6)	93 (67.4)	χ^2^ = 3.28	0.070
BMI, kg/m^2^	25.18 ± 2.80	24.30 ± 2.81	t = 1.99	0.049
Hypertension, n (%)	36 (64.3)	77 (55.8)	χ^2^ = 1.18	0.277
Diabetes mellitus, n (%)	13 (23.2)	24 (17.4)	χ^2^ = 0.88	0.350
Atrial fibrillation, n (%)	16 (28.6)	20 (14.5)	χ^2^ = 5.22	0.022
Prior stroke, n (%)	7 (12.5)	20 (14.5)	χ^2^ = 0.14	0.712
Current smoking, n (%)	23 (41.1)	56 (40.6)	χ^2^ = 0.00	0.946
Current alcohol use, n (%)	5 (8.9)	24 (17.4)	χ^2^ = 2.53	0.112
SBP, mmHg	164.98 ± 21.08	155.88 ± 21.33	t = 2.72	0.008
DBP, mmHg	97.23 ± 15.36	89.52 ± 12.55	t = 3.33	0.001
Heart rate, bpm	83.22 ± 11.99	78.32 ± 13.20	t = 2.50	0.014
GCS score	11 (10–13)	13 (12–14)	Z = −5.34	<0.001
NIHSS score	15 (8–17)	8 (4–13)	Z = 5.16	<0.001
Consciousness disturbance, n (%)	29 (51.8)	43 (31.2)	χ^2^ = 7.26	0.007
Prior antiplatelet therapy, n (%)	17 (30.4)	43 (31.2)	χ^2^ = 0.01	0.919
Prior anticoagulant therapy, n (%)	13 (23.2)	13 (9.4)	χ^2^ = 6.53	0.011

BMI, body mass index; DBP, diastolic blood pressure; GCS, Glasgow Coma Scale; HE, hematoma expansion; NIHSS, National Institutes of Health Stroke Scale; SBP, systolic blood pressure; bpm, beats per minute.

### 3.3 Laboratory findings between the hematoma expansion and non-expansion groups

The HE group exhibited higher admission leukocyte indices, with increased WBC count (9.74 ± 2.49 vs. 8.74 ± 1.98 × 10^9^/L; t = 2.70, *p* = 0.008) and neutrophil count (8.05 ± 2.32 vs. 5.90 ± 1.67 × 10^9^/L; t = 6.28, *p* < 0.001). Coagulation profiles differed significantly between groups, indicating more pronounced coagulation disturbance in the HE group, including a higher INR (1.22 ± 0.15 vs. 1.09 ± 0.10; t = 5.53, *p* < 0.001), longer PT (14.14 ± 1.31 vs. 13.20 ± 1.17 s; t = 4.70, *p* < 0.001) and APTT (34.62 ± 4.99 vs. 30.94 ± 4.25 s; t = 4.85, *p* < 0.001), as well as lower fibrinogen levels (3.10 ± 0.61 vs. 3.51 ± 0.71 g/L; t = −4.01, *p* < 0.001). Regarding biochemical parameters, serum creatinine and BUN were higher in the HE group (creatinine: 98.49 ± 25.49 vs. 85.99 ± 21.86 μmol/L; t = 3.22, *p* = 0.002; BUN: 8.71 ± 2.07 vs. 6.94 ± 1.89 mmol/L; t = 5.56, *p* < 0.001). Electrolyte analysis showed lower sodium and potassium levels in the HE group (sodium: 136.82 ± 3.72 vs. 139.52 ± 2.89 mmol/L; t = −4.87, *p* < 0.001; potassium: 4.02 ± 0.37 vs. 4.19 ± 0.44 mmol/L; t = −2.76, *p* = 0.007). Inflammatory and fibrinolysis-related biomarkers were also elevated in the HE group, including CRP (20.33 [9.78–32.66] vs. 12.14 [5.52–19.31] mg/L; Mann–Whitney U = 5,302, *p* < 0.001) and D-dimer (1.37 [0.79–2.43] vs. 0.56 [0.41–1.01] mg/L fibrinogen equivalent units; U = 5,995, *p* < 0.001). Conversely, hemoglobin concentration and platelet count did not differ significantly between groups (hemoglobin: t = −0.24, *p* = 0.813; platelet count: t = −0.19, *p* = 0.849; Table 2).

**Table 2:** Admission laboratory indices in the hematoma expansion and non-expansion groups.

Variable	HE (*n* = 56)	Non-HE (*n* = 138)	Test statistic	*p* value
White blood cell count (×10^9^/L)	9.74 ± 2.49	8.74 ± 1.98	t = 2.70	0.008
Neutrophil count (×10^9^/L)	8.05 ± 2.32	5.90 ± 1.67	t = 6.28	<0.001
Hemoglobin (g/L)	131.62 ± 16.55	132.20 ± 12.78	t = −0.24	0.813
Platelet count (×10^9^/L)	203.88 ± 54.87	205.53 ± 54.14	t = −0.19	0.849
International normalized ratio (—)	1.22 ± 0.15	1.09 ± 0.10	t = 5.53	<0.001
Prothrombin time (s)	14.14 ± 1.31	13.20 ± 1.17	t = 4.70	<0.001
Activated partial thromboplastin time (s)	34.62 ± 4.99	30.94 ± 4.25	t = 4.85	<0.001
Fibrinogen (g/L)	3.10 ± 0.61	3.51 ± 0.71	t = −4.01	<0.001
Plasma glucose (mmol/L)	8.78 ± 2.53	8.23 ± 1.75	t = 1.51	0.136
Serum creatinine (μmol/L)	98.49 ± 25.49	85.99 ± 21.86	t = 3.22	0.002
Blood urea nitrogen (mmol/L)	8.71 ± 2.07	6.94 ± 1.89	t = 5.56	<0.001
Sodium (mmol/L)	136.82 ± 3.72	139.52 ± 2.89	t = −4.87	<0.001
Potassium (mmol/L)	4.02 ± 0.37	4.19 ± 0.44	t = −2.76	0.007
C-reactive protein (mg/L)	20.33 (9.78–32.66)	12.14 (5.52–19.31)	U = 5,302	<0.001
D-dimer (mg/L FEU)	1.37 (0.79–2.43)	0.56 (0.41–1.01)	U = 5,995	<0.001

FEU, fibrinogen equivalent units; INR, international normalized ratio; PT, prothrombin time; APTT, activated partial thromboplastin time.

### 3.4 Baseline imaging features and hematoma growth

Baseline hematoma volume, as assessed by the ABC/2 method, was significantly larger in the HE group compared with the non-HE group (30.17 [17.89–58.43] vs. 14.62 [8.75–20.48] mL; U = 6,073, *p* < 0.001). This difference persisted on follow-up imaging, with the HE group showing a larger hematoma volume (47.75 [31.57–74.28] vs. 14.91 [9.14–20.51] mL; U = 7,040, *p* < 0.001). As expected, the HE group exhibited markedly greater hematoma growth, both in absolute volume (13.61 [9.76–19.45] vs. 0.45 [−0.65–1.27] mL; U = 7,728, *p* < 0.001) and relative percentage (45.60 [30.10–82.50] vs. 1.70 [−4.18–10.46]%; U = 7,424, *p* < 0.001). Hematoma location differed marginally between groups (χ^2^ = 7.80, *p* = 0.050), with a higher proportion of lobar hemorrhage in the HE group (50.0% vs. 32.6%) and a greater proportion of deep hemorrhage in the non-HE group (47.8% vs. 26.8%). Regarding NCCT morphological features, the HE group had significantly higher frequencies of irregular shape (67.9% vs. 39.9%; χ^2^ = 12.52, *p* < 0.001), blurred margin (53.6% vs. 32.6%; χ^2^ = 7.38, *p* = 0.007), and heterogeneous density (64.3% vs. 39.9%; χ^2^ = 9.55, *p* = 0.002). The black hole sign was also more prevalent in the HE group (39.3% vs. 10.1%; χ^2^ = 22.38, *p* < 0.001), whereas the blend sign did not differ significantly between groups (19.6% vs. 10.9%; χ^2^ = 2.64, *p* = 0.104). Additional radiological findings were more frequent in the HE group, including intraventricular hemorrhage (50.0% vs. 25.4%; χ^2^ = 11.03, *p* < 0.001), midline shift (46.4% vs. 13.0%; χ^2^ = 25.32, *p* < 0.001), and perihematomal edema (69.6% vs. 52.2%; χ^2^ = 4.97, *p* = 0.026; Table 3). Inter-rater reliability was excellent for hematoma volume measurement, with an ICC of 0.94 (95% CI, 0.89–0.99) for baseline hematoma volume and 0.93 (95% CI, 0.88–0.96) for follow-up hematoma volume. Agreement for categorical NCCT markers was substantial to excellent overall, with Cohen’s *κ* values ranging from 0.85 to 0.97 across the prespecified imaging signs.

**Table 3:** Baseline imaging characteristics and hematoma growth metrics in the hematoma expansion and non-expansion groups.

Variable	HE (*n* = 56)	Non-HE (*n* = 138)	Test statistic	*p* value
Baseline hematoma volume by ABC/2 (mL)	30.17 (17.89–58.43)	14.62 (8.75–20.48)	U = 6,073	<0.001
Follow-up hematoma volume (mL)	47.75 (31.57–74.28)	14.91 (9.14–20.51)	U = 7,040	<0.001
Absolute hematoma growth (mL)	13.61 (9.76–19.45)	0.45 (−0.65–1.27)	U = 7,728	<0.001
Relative hematoma growth (%)	45.60 (30.10–82.50)	1.70 (−4.18–10.46)	U = 7,424	<0.001
Hematoma location (overall), n (%)	—	—	χ^2^ = 7.80	0.050
Lobar, n (%)	28 (50.0)	45 (32.6)	—	—
Deep, n (%)	15 (26.8)	66 (47.8)	—	—
Brainstem, n (%)	7 (12.5)	13 (9.4)	—	—
Cerebellar, n (%)	6 (10.7)	14 (10.1)	—	—
Irregular shape, n (%)	38 (67.9)	55 (39.9)	χ^2^ = 12.52	<0.001
Blurred margin, n (%)	30 (53.6)	45 (32.6)	χ^2^ = 7.38	0.007
Heterogeneous density, n (%)	36 (64.3)	55 (39.9)	χ^2^ = 9.55	0.002
Blend sign, n (%)	11 (19.6)	15 (10.9)	χ^2^ = 2.64	0.104
Black hole sign, n (%)	22 (39.3)	14 (10.1)	χ^2^ = 22.38	<0.001
Intraventricular hemorrhage, n (%)	28 (50.0)	35 (25.4)	χ^2^ = 11.03	<0.001
Midline shift, n (%)	26 (46.4)	18 (13.0)	χ^2^ = 25.32	<0.001
Perihematomal edema, n (%)	39 (69.6)	72 (52.2)	χ^2^ = 4.97	0.026

ABC/2, ellipsoid approximation for hematoma volume (A × B × C / 2).

### 3.5 Univariable screening of potential factors associated with hematoma expansion

In univariable logistic regression analyses, greater neurological severity at admission was associated with higher odds of HE, as indicated by lower GCS scores (OR per 1-point increase: 0.60, 95% CI: 0.49–0.73; *p* < 0.001) and higher National Institutes of Health Stroke Scale scores (OR per 1-point increase: 1.17, 95% CI: 1.10–1.25; *p* < 0.001). Among clinical variables, older age, elevated admission blood pressure, presence of consciousness disturbance, atrial fibrillation, prior anticoagulant therapy, and higher heart rate were each associated with increased odds of HE (all *p* < 0.05). Conversely, several traditional vascular comorbidities and lifestyle factors, including hypertension, diabetes mellitus, prior stroke, smoking, and alcohol use, were not significantly associated with HE in univariable analyses (all *p* > 0.05). Laboratory screening identified significant associations between HE and elevated inflammatory indices, including WBC count and neutrophil count; coagulation abnormalities such as increased INR, prolonged PT and APTT, and decreased fibrinogen levels; elevated levels of CRP and D-dimer; markers of renal dysfunction, including increased serum creatinine and BUN; and lower concentrations of serum sodium and potassium (all *p* < 0.05). Imaging variables showed strong univariable associations with HE, including larger baseline hematoma volume and several adverse NCCT features such as midline shift, black hole sign, irregular shape, intraventricular hemorrhage, heterogeneous density, blurred margin, and perihematomal edema (all *p* < 0.05). Lobar hemorrhage was also associated with higher odds of HE compared with deep hemorrhage (*p* = 0.007), whereas the presence of a blend sign did not reach statistical significance (*p* = 0.109; Table 4).

**Table 4:** Univariable logistic regression screening of candidate factors for hematoma expansion.

Domain	Predictor (unit/increment)	OR	95% CI	*p* value
Clinical	GCS (per 1 point)	0.60	0.49–0.73	<0.001
	NIHSS (per 1 point)	1.17	1.10–1.25	<0.001
	Age (per 1 year)	1.13	1.06–1.20	<0.001
	DBP (per 10 mmHg)	1.53	1.20–1.95	<0.001
	Consciousness disturbance (yes vs. no)	2.37	1.26–4.48	0.008
	SBP (per 10 mmHg)	1.23	1.05–1.43	0.009
	Prior anticoagulant therapy (yes vs. no)	2.91	1.25–6.76	0.013
	Heart rate (per 10 bpm)	1.35	1.05–1.74	0.019
	Atrial fibrillation (yes vs. no)	2.36	1.12–4.99	0.025
	BMI (per 1 kg/m^2^)	1.12	1.00–1.25	0.050
	Men sex (men vs. women)	0.56	0.30–1.05	0.072
	Current alcohol use (yes vs. no)	0.47	0.18–1.21	0.118
	Hypertension (yes vs. no)	1.43	0.75–2.71	0.278
	Diabetes mellitus (yes vs. no)	1.44	0.67–3.07	0.351
	Prior antiplatelet therapy (yes vs. no)	1.22	0.61–2.46	0.571
	Prior stroke (yes vs. no)	1.16	0.53–2.57	0.712
	Current smoking (yes vs. no)	0.98	0.51–1.87	0.946
Laboratory	Neutrophils (per 1 × 10^9^/L)	1.84	1.49–2.27	<0.001
	INR (per 0.1)	2.25	1.66–3.03	<0.001
	BUN (per 1 mmol/L)	1.66	1.35–2.02	<0.001
	Sodium (per 1 mmol/L)	0.76	0.68–0.85	<0.001
	APTT (per 1 s)	1.20	1.11–1.30	<0.001
	PT (per 1 s)	1.95	1.45–2.62	<0.001
	CRP (per 10 mg/L)	1.47	1.20–1.82	<0.001
	Fibrinogen (per 1 g/L)	0.41	0.25–0.68	<0.001
	D-dimer (per 1 mg/L FEU)	1.58	1.21–2.05	<0.001
	Creatinine (per 10 μmol/L)	1.27	1.10–1.46	0.001
	WBC (per 1 × 10^9^/L)	1.24	1.07–1.44	0.004
	Potassium (per 0.1 mmol/L)	0.90	0.84–0.98	0.013
	Glucose (per 1 mmol/L)	1.15	0.98–1.34	0.083
	Hemoglobin (per 10 g/L)	0.97	0.78–1.21	0.790
	Platelets (per 10 × 10^9^/L)	0.99	0.94–1.05	0.847
Imaging	Baseline hematoma volume (per 10 mL)	1.89	1.50–2.37	<0.001
	Midline shift (yes vs. no)	5.78	2.81–11.89	<0.001
	Black hole sign (present vs. absent)	5.73	2.65–12.38	<0.001
	Irregular shape (present vs. absent)	3.19	1.65–6.14	<0.001
	Intraventricular hemorrhage (yes vs. no)	2.94	1.54–5.63	0.001
	Heterogeneous density (present vs. absent)	2.72	1.43–5.17	0.002
	Blurred margin (present vs. absent)	2.38	1.26–4.50	0.007
	Perihematomal edema (present vs. absent)	2.10	1.09–4.07	0.027
	Blend sign (present vs. absent)	2.00	0.86–4.69	0.109
	Lobar vs. deep location	2.74	1.32–5.70	0.007
	Brainstem vs. deep location	2.37	0.81–6.95	0.116
	Cerebellar vs. deep location	1.89	0.62–5.71	0.262

APTT, activated partial thromboplastin time; BMI, body mass index; BUN, blood urea nitrogen; CI, confidence interval; CRP, C-reactive protein; DBP, diastolic blood pressure; FEU, fibrinogen equivalent units; GCS, Glasgow Coma Scale; INR, international normalized ratio; NIHSS, National Institutes of Health Stroke Scale; OR, odds ratio; PT, prothrombin time; SBP, systolic blood pressure; WBC, white blood cell count.

### 3.6 Multivariable logistic regression: independent risk factors for hematoma expansion

After univariable screening, candidate predictors were entered into a multivariable logistic regression model. To mitigate potential multicollinearity, VIFs were evaluated before model fitting; all retained variables showed low collinearity (VIF range: 1.02–1.18), with none exceeding the predefined threshold of 5. To ensure model parsimony and minimize redundancy among highly correlated variables, the National Institutes of Health Stroke Scale (NIHSS) was selected to represent neurological severity (in place of the GCS), the INR was prioritized over PT and APTT, and neutrophil count was selected over total WBC count. A backward stepwise selection procedure was used to derive the final model. In the resulting multivariable logistic regression, older age, higher NIHSS score, elevated neutrophil count, increased INR, larger baseline hematoma volume, and the presence of the black hole sign and irregular hematoma shape were independently associated with increased odds of HE. Conversely, higher serum sodium concentration was independently associated with reduced odds of HE (Table 5). The final multivariable model retained eight predictor parameters (age, NIHSS score, neutrophil count, INR, sodium, baseline hematoma volume, black hole sign, and irregular shape) for 56 HE events, corresponding to an EPV of 7.0 (Supplementary Table S1).

**Table 5:** Independent predictors of hematoma expansion in multivariable logistic regression.

Predictor (increment)	Adjusted OR	95% CI	*p* value
Age (per 1 year)	1.13	1.02–1.26	0.025
NIHSS (per 1 point)	1.21	1.08–1.35	<0.001
Neutrophil count (per 1 × 10^9^/L)	1.91	1.34–2.73	<0.001
INR (per 0.1 increase)	1.70	1.08–2.69	0.023
Sodium (per 1 mmol/L)	0.76	0.64–0.91	0.003
Baseline hematoma volume by ABC/2 (per 10 mL)	2.01	1.34–3.02	<0.001
Black hole sign (present vs. absent)	7.34	1.87–28.77	0.004
Irregular shape (present vs. absent)	5.57	1.67–18.63	0.005

INR was scaled per 0.1-unit increment in the model; thus, its coefficient and adjusted odds ratio correspond to each 0.1 increase in INR.

### 3.7 Prediction model development and risk stratification

**Figure 1:**
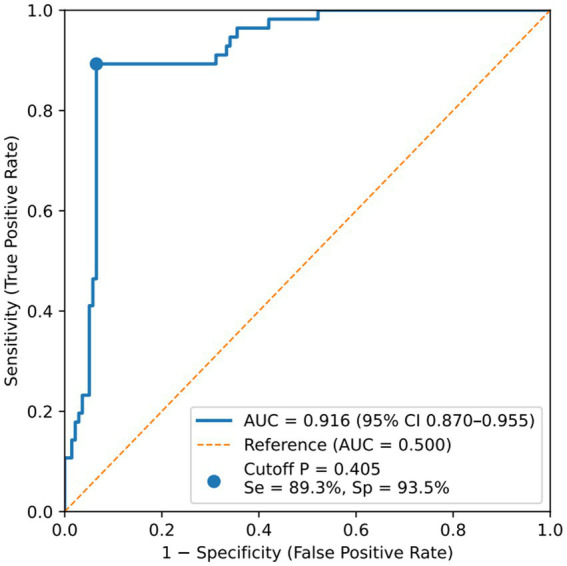
Receiver operating characteristic (ROC) curve for the hematoma expansion (HE) prediction model. The ROC curve demonstrates the discriminative performance of the multivariable logistic regression model for predicting HE in elderly patients with spontaneous intracerebral hemorrhage. The area under the curve (AUC) was 0.916 (95% confidence interval [CI], 0.870–0.955). The optimal probability cutoff was 0.405, yielding a sensitivity of 89.3% and a specificity of 93.5% (blue marker). The dashed diagonal line indicates the no-discrimination reference (AUC = 0.500).

A prediction model for HE was developed using multivariable logistic regression, incorporating all independent predictors identified in the final adjusted model: age, NIHSS score, neutrophil count, INR, serum sodium level, baseline hematoma volume measured by the ABC/2 method, presence of the black hole sign, and irregular hematoma shape. The estimated probability of HE for an individual patient was calculated from the fitted logistic equation: logit(P[HE]) = 11.894 + 0.122 × Age (years) + 0.191 × NIHSS + 0.647 × Neutrophils (×10^9^/L) + 0.533 × (INR/0.1) − 0.269 × Sodium (mmol/L) + 0.699 × (Baseline hematoma volume/10 mL) + 1.993 × Black hole sign (present) + 1.718 × Irregular shape (present), where logit(P) = ln(P/(1 − P)). Binary imaging variables were coded as 1 if present and 0 if absent. In the development dataset, the model showed an apparent AUC of 0.916. Internal validation using 1,000 bootstrap resamples showed modest optimism, with a mean optimism of 0.025 and an optimism-corrected AUC of 0.891 (Supplementary Table S2). Calibration analysis yielded a bootstrap-corrected calibration intercept of −0.01 and a calibration slope of 0.87, indicating good calibration with mild overfitting. The apparent and optimism-corrected Brier scores were 0.104 and 0.111, respectively. Using the optimal probability cutoff derived from the ROC curve (*p* = 0.405), the model achieved a sensitivity of 89.3%, a specificity of 93.5%, a positive predictive value of 84.7%, and a negative predictive value of 95.6% (Figure 1). DCA showed that the model provided greater net benefit than the default treat-all and treat-none strategies across selected threshold probabilities of 10 to 50% (Supplementary Table S3). For clinical application, patients were stratified into three predefined risk categories according to their predicted probability of HE: low risk (<10%), intermediate risk (10–30%), and high risk (>30%).

### 3.8 Subgroup analyses

Prespecified subgroup analyses evaluated whether the discriminative performance of the HE prediction model varied across clinically relevant strata, including hemorrhage location (lobar, deep, brainstem, cerebellar), pre-intracerebral hemorrhage (ICH) antithrombotic exposure (anticoagulant use vs. none; antiplatelet use vs. none), and follow-up NCCT time window (≤6 h, 6–24 h, 24–48 h). Discrimination was assessed using ROC analysis within each subgroup. The model demonstrated high discriminatory performance across all hemorrhage locations, with AUC values of 0.977 for lobar hemorrhage (*n* = 73; HE = 28), 0.951 for deep hemorrhage (*n* = 81; HE = 15), 0.868 for brainstem hemorrhage (*n* = 20; HE = 7), and 0.917 for cerebellar hemorrhage (*n* = 20; HE = 6). Similarly, strong performance was observed in antithrombotic exposure subgroups: anticoagulant use (AUC = 0.976, *n* = 26), no anticoagulant use (AUC = 0.954, *n* = 168), antiplatelet use (AUC = 0.975, *n* = 60), and no antiplatelet use (AUC = 0.947, *n* = 134). Across follow-up CT timing strata, the model maintained robust discrimination: ≤6 h (AUC = 0.964, *n* = 95), 6–24 h (AUC = 0.955, *n* = 78), and 24–48 h (AUC = 0.926, *n* = 21). Formal interaction testing revealed no statistically significant effect modification of model discrimination by hemorrhage location (P_interaction = 0.287), follow-up CT time window (P_interaction = 0.933), anticoagulant use (P_interaction = 0.153), or antiplatelet use (P_interaction = 0.683). However, given the limited sample sizes in the posterior fossa subgroups (brainstem and cerebellar hemorrhages), these estimates should be interpreted with caution.

### 3.9 Sensitivity analyses

Sensitivity analyses were conducted to assess the robustness of the model’s discriminative performance to alternative definitions of HE and to the exclusion of extreme values. When HE was redefined using the absolute volume criterion alone (increase >6 mL), the AUC remained unchanged at 0.916, consistent with the primary model. Similarly, a composite definition incorporating a higher relative growth threshold (absolute increase >6 mL or relative increase >50%) yielded the same AUC of 0.916. Under a more stringent criterion (absolute increase >12 mL or relative increase >33%), the model continued to demonstrate strong discrimination (AUC = 0.940). When the relative growth criterion alone (increase >33%) was used, the AUC decreased to 0.879, potentially reflecting reduced statistical power due to fewer HE events under this definition. Further analyses excluding extreme values, defined as the upper 5th percentile of baseline hematoma volume and/or absolute hematoma growth, further confirmed model robustness. The AUC remained stable after excluding extreme baseline volumes (AUC = 0.951), extreme growth values (AUC = 0.954), and both simultaneously (AUC = 0.949). These findings support the consistency and generalizability of the model’s discriminative capacity across varying definitions of HE and patient subgroups.

## 4 Discussion

In this retrospective cohort of elderly patients with sICH, HE appeared to reflect the combined effects of baseline hemorrhage burden, neurological severity, coagulation status, inflammatory activation, and adverse NCCT morphology. Building on these domains, we developed a parsimonious and clinically interpretable prediction model that integrated routinely available admission variables across neurological, laboratory, and imaging assessments. The novelty of this study lies in its focus on older adults, a clinically vulnerable population in whom early HE prediction remains insufficiently characterized, and in its emphasis on variables that can be obtained rapidly in routine stroke care without reliance on advanced imaging, radiomics, or computationally intensive workflows. The model showed strong discrimination in the derivation cohort and remained stable across prespecified subgroups and sensitivity analyses, suggesting that it captures a clinically meaningful signal of early hematoma instability in elderly sICH. From a practical perspective, this approach may facilitate early bedside risk stratification, support decisions regarding monitoring intensity and repeat neuroimaging, and help identify patients who may benefit from closer neurocritical surveillance or more proactive hemostatic management (Wang and Cui, 2025; Kim et al., 2026). Overall, our findings extend the current HE literature by providing an elderly-specific and pragmatically applicable framework for early risk assessment in acute sICH.

The present findings suggest that HE in elderly patients with sICH is not driven by a single domain but reflects the convergence of clinical severity, biological vulnerability, and baseline structural instability of the hematoma. Older age remained independently associated with HE even after adjustment, suggesting that age in this setting is more than a demographic descriptor; it likely captures cumulative frailty, impaired vascular integrity, reduced homeostatic reserve, and a greater burden of comorbidity and antithrombotic exposure that are not fully reflected by any single measured variable (Yu and Alexander, 2024; Sheth et al., 2024). The independent effect of the NIHSS further supports the view that early neurological severity is not merely a consequence of the initial hemorrhage but may also identify patients with more active or unstable bleeding (Wu X. et al., 2024; Cummock et al., 2023). From a biological perspective, the strong associations between neutrophil count and INR with HE are clinically plausible (Ducroux et al., 2023; Makharia et al., 2024). Elevated neutrophils may reflect an acute inflammatory response accompanying tissue injury and endothelial dysfunction, whereas INR represents impaired hemostatic competence and therefore a reduced ability to limit ongoing hemorrhage (Kazaryan et al., 2024; Rodriguez-Luna et al., 2025). The inverse association with serum sodium is also noteworthy. Rather than implying a direct causal effect, lower sodium may serve as an integrated marker of acute physiological stress, systemic illness severity, or disturbed neurohumoral regulation in the hyperacute phase of ICH (Wu Q. et al., 2024; Zou et al., 2025). These interpretations are broadly in line with prior research indicating that HE risk is influenced by inflammatory activity, coagulation status, and the severity of the initial insult (Qian et al., 2024). Collectively, our results indicate that early HE risk assessment in elderly sICH should not rely solely on traditional clinical observation or isolated laboratory abnormalities, but should be based on a multidimensional framework that incorporates neurological examination, coagulation and inflammatory markers, and early imaging characteristics to identify patients in whom bleeding may still be dynamically evolving (Cindea et al., 2025a; Ducroux et al., 2023; Rodriguez-Luna et al., 2025).

The imaging results further reinforce the concept that baseline NCCT can provide meaningful information about hematoma instability beyond simple confirmation of hemorrhage (Guo and Zou, 2024; Loggini et al., 2025). A larger baseline hematoma volume was independently associated with HE, consistent with the idea that a greater initial bleeding burden may reflect either more severe vessel injury or a larger substrate for continued enlargement. More importantly, the black hole sign and irregular hematoma shape remained robust predictors after multivariable adjustment, suggesting that these markers convey information not fully captured by hematoma size or clinical severity alone. Morphologically, both signs may indicate internal heterogeneity of clot formation, uneven bleeding dynamics, or incomplete tamponade, all of which are compatible with an unstable hematoma phenotype (Morotti et al., 2024; Morotti et al., 2025). The strong discriminative performance of the final model, with an AUC of 0.916 and high sensitivity and specificity at the selected threshold, indicates that routinely available admission variables can provide substantial prognostic separation in this elderly population. Simultaneously, the model’s performance across hemorrhage location strata, antithrombotic exposure subgroups, and different follow-up CT windows suggests that its predictive signal is not confined to a narrow clinical subset (Cottarelli et al., 2025). The consistency of discrimination across alternative HE definitions and after excluding extreme values further supports the robustness of the overall pattern, particularly because HE classification can vary with operational thresholds (Tanioka et al., 2024; Yalcin et al., 2024; Lee et al., 2024). Taken together, these results suggest that a pragmatic model integrating age, NIHSS, neutrophil count, INR, sodium, hematoma volume, and key NCCT markers may help refine early bedside risk stratification for elderly patients with sICH. Clinically, such an approach may be useful for prioritizing closer monitoring, optimizing the timing of repeat imaging, and identifying patients for whom more aggressive hemostatic or neurocritical care strategies warrant particular consideration (Stretz et al., 2023).

Compared with prior HE prediction studies, our findings support the broader view that HE can be predicted using combinations of early clinical and imaging markers, while also highlighting the value of a more pragmatic, elderly-focused framework. Huynh et al. (2015) validated the 9-point and 24-point HE scores and derived the PREDICT A/B scores in a multicenter cohort using CTA-based data. They showed that established predictors such as neurological severity, coagulopathy, and hemorrhage burden have acceptable discriminative utility, although calibration and performance varied across models. Our results are directionally consistent with these observations, as age, neurological severity, INR-related coagulation disturbance, and baseline hematoma burden were likewise associated with HE. However, our study extends this line of study by focusing specifically on elderly patients with sICH and by constructing a model based entirely on routinely available admission variables and standard NCCT findings, without requiring CTA. Similarly, Lim et al. (2019) externally validated the PREDICT, 9-point, and BRAIN scores in an Asian cohort and found that PREDICT performed best, although overall discrimination and calibration remained only moderate. Their study underscores the importance of formal validation and benchmarking, whereas our study addresses a different but complementary question, namely, whether an elderly-specific derivation model based on accessible bedside variables can achieve clinically useful discrimination. Tanioka et al. (2022) showed that machine learning approaches could outperform conventional HE scores, suggesting that greater computational complexity may improve predictive accuracy. Conversely, our study prioritized interpretability and implementation feasibility over algorithmic complexity and still achieved strong discrimination using conventional multivariable modeling. Taken together, the novelty of the present study does not primarily lie in identifying entirely new individual predictors, but in deriving an elderly-specific, clinically deployable HE prediction model based on routinely available admission clinical, laboratory, and standard NCCT variables. In this sense, our research provides an incremental but practical contribution to early risk stratification in elderly sICH by emphasizing feasibility, transparency, and bedside applicability in a clinically vulnerable population.

This study evaluated HE predictors in an elderly-specific cohort and integrated clinical severity, laboratory indices, and interpretable non-contrast CT signs into a single logistic framework. The imaging component combined standardized volumetry (ABC/2) with morphological features amenable to routine interpretation, enhancing potential clinical transferability. The model was further examined in prespecified subgroup and sensitivity analyses, providing information on robustness to imaging time windows and alternative HE definitions. In this study, several limitations should be considered. First, the retrospective design may have introduced selection bias, particularly because inclusion required both baseline and follow-up NCCT within the predefined window and the presence of sufficiently complete clinical, laboratory, and imaging data. Local admission patterns, imaging practices, and treatment pathways may therefore have influenced both case selection and model performance. Second, although the model showed strong apparent discrimination and remained stable in subgroup and sensitivity analyses, external validation was not performed, and generalizability across institutions, imaging workflows, and patient populations remains uncertain. We also did not perform formal head-to-head benchmarking against existing HE prediction models. Third, to preserve bedside feasibility, the model relied on routinely available admission variables and standard NCCT markers and did not incorporate CTA markers or more advanced quantitative imaging features, which may provide additional predictive value in some settings. Fourth, hematoma multiplicity within the same patient was not modeled as a separate lesion-level factor and may have influenced volumetric and imaging-based assessment in a subset of cases. Finally, the association between serum sodium and HE should be interpreted with caution, as its mechanistic significance remains uncertain. Furthermore, the relatively high apparent AUC should be interpreted with caution. The modest events-per-variable ratio, the decrease from the apparent to the optimism-corrected AUC, and a calibration slope below 1.0 all suggest some degree of model optimism and mild overfitting in the derivation cohort. Accordingly, the present model should be regarded as a derivation model that requires confirmation in independent external datasets before broader clinical application. Future studies should include prospective multicenter cohorts, independent external validation, standardized handling of multiple hemorrhagic foci, and evaluation of whether dynamic treatment-related variables or selected advanced imaging markers improve prediction beyond the current admission-based framework.

## 5 Conclusion

In elderly patients with sICH, HE may be associated with older age, higher NIHSS score, elevated neutrophil count, higher INR, lower serum sodium, larger baseline hematoma volume, and adverse non-contrast CT morphology, particularly the black hole sign and irregular shape. A multivariable logistic model integrating these predictors might provide high discriminative performance in identifying patients at increased risk of HE.

## Data Availability

The raw data supporting the conclusions of this article will be made available by the authors, without undue reservation.
