# Supplementary material for: Risk factors and development of a prediction model for hematoma expansion in elderly patients with spontaneous intracerebral hemorrhage
Source: Front Aging Neurosci. 2026 May 28;18:1791268. doi: 10.3389/fnagi.2026.1791268 (PMC13261806; doi:10.3389/fnagi.2026.1791268)
Supplement: Supplementary file 1 [file Table_1.docx]

Supplementary Table S1. Model development sample size considerations and apparent performance

| Item | Value |
| --- | --- |
| Total sample size, n | 194 |
| HE events, n | 56 |
| Non-HE patients, n | 138 |
| Outcome prevalence, % | 28.9 |
| Predictor parameters retained in the final model, n | 8 |
| EPV | 7 |
| Apparent AUC | 0.916 |

HE, hematoma expansion; EPV, events per variable; AUC, area under the receiver operating characteristic curve. EPV was calculated as the number of hematoma expansion events divided by the number of predictor parameters retained in the final multivariable model, excluding the intercept. Apparent AUC refers to model discrimination estimated in the development dataset without optimism correction.

Supplementary Table S2. Bootstrap internal validation and calibration performance of the hematoma expansion prediction model

| Domain | Metric | Apparent estimate | Optimism | Optimism-corrected estimate | Interpretation |
| --- | --- | --- | --- | --- | --- |
| Discrimination | AUC | 0.916 | 0.025 | 0.891 | Good discrimination after correction |
| Calibration | Calibration intercept | 0.000 | 0.010 | −0.01 | Minimal systematic overprediction/underprediction |
| Calibration | Calibration slope | 1.000 | 0.130 | 0.870 | Mild overfitting |
| Overall performance | Brier score | 0.104 | 0.007 | 0.111 | Acceptable overall prediction error |
| Internal validation | Bootstrap resamples, n | 1000 | — | — | Entire model-development process repeated |

AUC, area under the receiver operating characteristic curve. The AUC of 0.916 is not the internally validated AUC, but the apparent AUC estimated in the development dataset; although it indicates good discrimination, it may be optimistic. Internal validation was performed using 1,000 bootstrap resamples, with repetition of the entire model-development process in each resample. The optimism-corrected AUC was calculated by subtracting the mean bootstrap optimism from the apparent AUC. Calibration was summarized using the bootstrap-corrected calibration intercept and calibration slope, and overall performance was assessed using the apparent and optimism-corrected Brier scores.

Supplementary Table S3. Representative net benefit values from decision curve analysis at selected threshold probabilities

| Threshold probability | Model net benefit | Treat-all net benefit | Treat-none net benefit |
| --- | --- | --- | --- |
| 0.1 | 0.246 | 0.210 | 0.000 |
| 0.2 | 0.188 | 0.111 | 0.000 |
| 0.3 | 0.152 | −0.016 | 0.000 |
| 0.4 | 0.123 | −0.185 | 0.000 |
| 0.5 | 0.098 | −0.422 | 0.000 |

Net benefit was calculated according to decision curve analysis (DCA). Positive net benefit greater than both the treat-all and treat-none strategies indicates potential clinical utility at the corresponding threshold probability.
